# Different recommendations for empiric first-choice antibiotic treatment of uncomplicated urinary tract infections in Europe

**DOI:** 10.3109/02813432.2013.844410

**Published:** 2013-12

**Authors:** Josephine McQuiston Haslund, Marianne Rosborg Dinesen, Anni Brit Sternhagen Nielsen, Carl Llor, Lars Bjerrum

**Affiliations:** ^1^Section and Research Unit of General Practice, Department of Public Health, University of Copenhagen, Denmark; ^2^University Rovira I Virgili, Tarragona, Spain

**Keywords:** Antibiotics, antimicrobial resistance, Denmark, general practice, primary health care, recommendations, uncomplicated urinary tract infection

## Abstract

**Objective:**

Uncomplicated urinary tract infection (uUTI) is a common reason for antibiotic treatment in primary health care. Due to the increasing prevalence of antibiotic-resistant uropathogens it is crucial to use the most appropriate antibiotics for first-choice empiric treatment of uUTI. Particularly, it is important to avoid antibiotics associated with a high rate of antimicrobial resistance. This study compares national recommendations from six European countries, investigating recommendations for first-choice antibiotic therapy of uUTI.

**Setting:**

General practice in six European countries.

**Method:**

Searches were undertaken on PubMed, the Cochrane Library databases, Google, and Google Scholar. Recommendations from different geographical regions in Europe were investigated: Northern Europe (Denmark, Sweden), Western Europe (Scotland), Central Europe (Germany), Southern Europe (Spain), and Eastern Europe (Croatia).

**Results:**

The six countries recommended seven different antibiotics. Five countries recommended more than one antibiotic as first-choice treatment. Half of the countries recommended antibiotics associated with a high rate (> 10–20%) of resistant *E. coli.* All countries recommended at least one antibiotic associated with a low (< 5%) resistance rate.

**Discussion:**

The differences in first-choice treatment of uUTI could not be explained by differences in local bacterial aetiology or by different patterns of antimicrobial resistance. Despite resistance rates exceeding 10–20%, sulphamethizole, trimethoprim. or fluoroquinolones were recommended in half of the countries.

**Conclusion:**

Within the European countries there are considerable differences in recommendations for empiric first-choice antibiotic treatment of uUTI. In order to reduce the increasing antimicrobial resistance in Europe, it is important to agree on the most appropriate antibiotics for empiric treatment of uUTI.

The increasing antimicrobial resistance in Europe makes it important to agree on the most appropriate antibiotics for empiric first-choice treatment of uncomplicated urinary tract infections.

- Recommendations for empiric antibiotic treatment of uncomplicated urinary tract infection differ considerably between six European countries.
- Half of the countries recommend antibiotics for which the resistance rate of *E. coli* is more than 10–20%.
- Use of antibiotics with resistance rates exceeding 10–20% is associated with an increased risk of treatment failure and selection of resistant strains.

## Introduction

Uncomplicated urinary tract infection (uUTI) is one of the most common infections in primary care [1]. Some 10% of all women will experience an episode of uUTI within a year. About 20% of all antibiotic prescriptions issued by GPs are for patients with uUTI. Patients with uUTI are often treated empirically without preceding culture or susceptibility testing, and the GP's choice of antibiotics typically depends on traditions and national treatment recommendations. Without treatment, the infection is often self-limiting over time and only seldom associated with serious complications [2,3]. However, in most cases, antibiotic treatment leads to symptom relief and bacterial eradication within a few days, making antibiotic treatment relevant [4,5].

Due to the increasing prevalence of antibiotic- resistant bacteria, particularly the extended spectrum beta-lactamase (ESBL) producing gram-negative bacteria, it is crucial to avoid antibiotic overprescribing. Particularly, it is important to avoid antibiotics that provoke the emergence of antimicrobial resistance, such as the so-called critically important antibiotics [6]. The pattern of microbial resistance and the use of antibiotics vary considerably between countries [7]. During the last decade the resistance rate of *E. coli* to fluoroquinolone has increased, particularly in the Southern European countries. In the Nordic countries an increasing resistance rate of *E. coli* to sulphamethizole and ampicillin is emerging [8]. In all European countries, *E. coli* is the most frequent aetiology of uUTI, accounting for 80–90% of all cases [9].

According to the European Survey of Antibiotic Consumption (ESAC), resistant strains are responsible for an attribute mortality of about 25 000 Europeans yearly. A considerable part of this increased mortality is caused by complications of urinary tract infections [10].

To reduce the selection of resistant bacteria, empiric prescribing of broad-spectrum antibiotics should be avoided in patients with trivial and uncomplicated infections, such as uUTI. Furthermore, critically important antibiotics, such as fluoroquinolones, should be restricted to the most severe infections and always be preceded by a susceptibility test. Moreover, antibiotics where resistance rates of the most frequent uropathogens exceed 10–20% should be avoided due to an increased risk of treatment failure and complications [11–13].

In order to reduce the increased risk of complications related to infections with resistant bacteria it is crucial to choose the most appropriate antibiotic for first-choice empiric treatment for uUTI. The aim of this study was to compare recommendations for empiric first-choice empiric antibiotic treatment of uUTI in a selected number of countries, from different geographical areas of Europe.

## Material and methods

We searched (September 2011) for literature in the PubMed and Cochrane Library Databases, Google, and Google Scholar. The following search terms were used: *urinary tract infection, treatment, clinical guideline, recommendations*, and *antimicrobial resistance.*

Recommendations were included if they met the following criteria:

- National recommendation for empiric antibiotic treatment of uUTI in primary health care.
- Agreement about the definition of uUTI: *Acute urinary tract infection in a premenopausal, non-pregnant woman, with no anatomical and functional abnormalities of the urogenital tract, no recent instrumentation of the urinary tract, and no comorbidity.*
- Language restriction to: English, Spanish, German, Norwegian, Swedish, and Danish.

Recommendations for treatment of recurrent UTI, complicated UTI (men, children, and patients with comorbidity), postmenopausal and post-surgery UTI were excluded.

Recommendations from six countries in five different geographical areas of Europe were included:

- Northern Europe: Denmark [14–17], Sweden [18];
- Western Europe: Scotland [19];
- Eastern Europe: Croatia [20];
- Southern Europe: Spain [21];
- Central Europe: Germany [22].

## Results

Recommendations for first-choice empiric antibiotic treatment of patients with uUTI are given in Table I. In total, seven preparations from six different groups of antimicrobials were recommended [23].

- beta-lactams;
- sulphonamide;
- trimethoprim;
- fluoroquinolone;
- phosphonic acid derivative;
- furan derivative.

**Table I.** Recommendations for first choice antibiotic treatment of uUTI in Europe.

	Nitrofurantoin	Pivmecillinam	Trimethoprim	Sulphamethizole	Fluoroquinolone	Fosfomycine/ trometamol	Amoxicillin/ clavulanic acid
Croatia^a^	√						
Denmark^b^		√	√	√			
Germany^c^	√	√				√	
Scotland^d^	√		√				
Spain^e^	√				√	√	√
Sweden^f^	√	√					

^a^Skerk V, Andrasevic AT, Andrasevic S, Susic E, Dzepina AM, Madaric V et al. [ISKRA guidelines on antimicrobial treatment and prophylaxis of urinary tract infections—Croatian national guidelines]. Lijec Vjesn 2009;131(5–6):105–118.

^b^IRF, Institut for Rationel Farmakoterapi [internet]. Antibiotika til systemisk brug (Institute for Rational Pharmacotherapy, Antibiotics for Systemic Use). 12-2-2009. [cited 1–5-2012]. Available from: http://www.irf.dk/dk/rekommandationsliste/infektionssygdomme/antibiotika_systemisk_brug/test_sulfonamider_og_trimethoprim_j01e.htm.

Basislisten 2011 [internet]. Basislisten, lægens værktøj til rationel medicinordination. (The Basic list, the doctor’s tool for rational drug prescribing). [cited 3-2-2012]. Available from: https://www.sundhed.dk/content/cms/15/4915_2012-basislisten.pdf

Helweg-Larsen J, Frimodt-Moller N, Nielsen H, Dahl Knudsen J, Holme P. Antibiotika (systemisk brug), baggrundsnotat, 2009. (Antibiotics (systemic use), background notes, 2009). [internet]. [cited 12-9-2009]. Available from: http://www.irf.dk/dk/rekommandationsliste/baggrundsnotater/infektionssygdomme/antibiotika_systemisk_brug.htm#Uro-genitale_infektioner.

Gahrn-Hansen B, Kolmos HJJ. Antibiotikapolitik og behandling af hyppigt forekommende infektioner i almen praksis. (Policy of antibiotics and treatment of common infections in general practice). Institute for Rational Pharmacotherapy. Danish Medicines Agency. 2001;7.

^c^Wagenlehner FM, Schmiemann G, Hoyme U, Funfstuck R, Hummers-Pradier E, Kaase M et al. Nationale S3-Leitlinie, unkomplizierte harnwegsinfektionen. Empfehlungen zu terapie und management unkomplizierter bakterieller ambulant erworbener harnwegsinfektionen bei erwachsenen patienten. (S3-guideline for uncomplicated urinary tract infections - treatment guidelines compliance). Med Monatsschr Pharm 2011;34(5):164–168.

^d^Scottish Intercollegiate Guideline Network. Management of suspected bacterial urinary tract infection in adults. A national clinical guideline. 2012; 7–12.

^e^Rabanaque G, Romera Á, Domingo C, Herrera C, Plana A, Sánchez J. Infecciones del tracto urinario. (Urinary tract infections). Manual de enfermedades infecciones en atención primaria. (Handbook of infectious diseases in primary helth care). Barcelona: 2010:153–181.

^f^André M, Ahlqvist-Rastad J, Beermann B. Nedre urinvägsinfektion (UVI) hos kvinnor - Behandlingsrekommendation. (Lower urinary tract infection (UTI) in women - Treatment recommendation). Läkemedelsverket 2007;2:8–15.

Most countries [15–19,21,22] included more than one antibiotic as first-choice recommendation for uUTI, but none of the preparations was recommended by all countries. Nitrofurantoin was included as first-choice antibiotic in five countries and pivmecillinam in three countries. Spain [21] was the only country including fluoroquinolone and amoxicillin/clavulanic acid as first-choice recommendation, and Denmark was the only country recommending sulphamethizole [14–17].

## Discussion

The national recommendations for first-choice empiric antibiotic treatment of uUTI varied considerably between the six European countries. Nitrofurantoin and pivmecillinam were the preparations most agreed upon; they were recommended in five and three countries, respectively. Spain was the only country recommending fluoroquinolones and amoxicillin/clavulanic acid as first-choice antibiotic for uUTI. Denmark was the only country recommending sulphamethizole.

Before conclusion, some limitations have to be taken into account. We have no information about the adherence to the recommendations in the different countries, and we are not aware of potential regional recommendations that may have overruled the national recommendations. Furthermore, we may have overlooked guidelines that were not published in peer-reviewed journals or available on the internet. This is, however, not likely since two of the authors (JMH, MRD) searched separately. For each of the included countries we tried to identify the newest guideline available. However, two guidelines were more than five years old. Older guidelines may not have accounted for the increased prevalence of resistant strains during recent years. Furthermore, we did not include information on the availability of different kinds of antibiotics in the countries included. Some antibiotics may not be available in all the countries and therefore would not be included in the guideline. For example, pivmecillinam is at the moment not available in Spain.

The differences found between the six countries are considered to be minimum estimates of the real differences in recommendations for antibiotic treatment of uUTI between the European countries. Inclusion of more European countries might have led to a higher number of different recommendations for first-choice treatment of uUTI. Antibiotic resistance rates differ considerably between the countries (Table II). In all six countries *E. coli* has a high resistance rate against sulphamethizole (22–37%) and trimethoprim (15–32%), and a low resistance rate against nitrofurantoin (< 5%), mecillinam (1–5%) and fosfomycine (< 2%) [14–22,24]. The rate of resistance to fluoroquinolones, in urine gathered from the primary care sector, varies considerably between the European countries with the lowest rate in Scotland (1%) and the highest rate in Spain (24%) [9,21,24]. Results of the susceptibility pattern of uropathogens from urine samples submitted from primary care to a microbiology department are, however, highly dependent on the groups of patients from which the urine samples are collected. We have limited knowledge regarding the bacterial resistance pattern in patients with uncomplicated UTI because these patients are most often treated without a preceding urine culture and susceptibility test. However, in patients suspected to suffer from a complicated UTI the GP will often ask for a culture and susceptibility test before making a decision about antibiotic treatment. Therefore, results of the antimicrobial resistance pattern may be biased by a high number of uropathogenic bacteria from complicated UVI.

**Table II.** Antimicrobial resistance (%) for *E. coli* in urine samples from patients with urinary tract infections, in six European countries.

	Nitrofurantoin (%)	Pivmecillinam (%)	Fosfomycine (%)	Sulfamethizole (%)	Trimethroprim (%)	Ciprofloxacin (%)	Amoxicillin/ clavulanic acid (%)
Croatia^a^ (2009)	2	–	–	24*	24*	10	4
Denmark^b^ (2009)	0–5	4–5	–	35–38	10–28	11	–
Germany^c^ (2006)	1–2	1–2	< 1	25–26*	25–26*	3–4	1–2
Scotland^d**^ (2000)	0	1–2	< 1	36	16–17	1–2	3–4
Spain^e^ (2006)	3–4	4	1–2	32*	32*	23.9	8
Sweden^f^ (2007)	1	3	1	15*	15*	5–10	25

Notes: * = resistance to trimethroprim/sulfamethizole. – = no data.

^a^Skerk V, Andrasevic AT, Andrasevic S, Susic E, Dzepina AM, Madaric V et al. [ISKRA guidelines on antimicrobial treatment and prophylaxis of urinary tract infections—Croatian national guidelines]. Lijec Vjesn 2009;131(5–6):105–118.

^b^Danmap 2009 [internet]. Use of antimicrobial agents and occurrence of antimicrobial resistance from food animals, fruits and humans in Denmark. [cited 1–6-2011.] Available from: www.danmap.org. 2009.

IRF, Institut for Rationel Farmakoterapi [internet]. Antibiotika til systemisk brug (Institute for Rational Pharmacotherapy, Antibiotics for Systemic Use).12-2-2009. [cited 1–5-2012]. Available from: http://www.irf.dk/dk/rekommandationsliste/infektionssygdomme/antibiotika_systemisk_brug/test_sulfonamider_og_trimethoprim_j01e.htm.

^c^Naber K, Schito G, Botto H, Palou J, Mazzei T. Surveillance study in Europe and Brazil on Clinical Aspects and antimicrobial Resistance Epidemiology in females with cystitis (ARESC): Implications for Empiric Therapy. European Urology 2008:54:1164–1178.

^d^Kahlmeter G. The ECO.SENS Project: a prospective, multinational, multicentre epidemiological survey of the prevalence and antimicrobial susceptibility of urinary tract pathogens—interim report. J Antimicrob Chemother 2000;46 Suppl 1:15–22.

^e^Rabanaque G, Romera Á, Domingo C, Herrera C, Plana A, Sánchez J. Infecciones del tracto urinario. (Urinary tract infections). Manual de enfermedades infecciones en atención primaria. (Handbook of infectious diseases in primary helth care). Barcelona: 2010:153–181.

Naber K, Schito G, Botto H, Palou J, Mazzei T. Surveillance study in Europe and Brazil on Clinical Aspects and antimicrobial Resistance Epidemiology in females with cystitis (ARESC): Implications for Empiric Therapy. European Urology 2008:54:1164–1178.

^f^André M, Ahlqvist-Rastad J, Beermann B. Nedre urinvägsinfektion (UVI) hos kvinnor - Behandlingsrekommendation. (Lower urinary tract infection (UTI) in women - Treatment recommendation). Läkemedelsverket 2007;2:8–15.

The differences found in the recommendations for first-choice treatment of uUTI cannot be supported by different patterns of bacterial aetiology or a different resistance pattern for *E. coli*. In all countries, *E. coli* accounts for the majority (70–90%) of uUTI [14–22] and *S. saprophyticus* is the second most common uropathogen, accounting for 2–11% of uUTI, and most frequent in young women [4,9].

The reasons for the diverging recommendations may be due to different price policies, different availability, and/or different traditions. The potential influence of such factors deserves further investigation.

All six countries recommended one or more antibiotics with a low risk of *E. coli* resistance such as nitrofurantoin, mecillinam, and fosfomycine as first-choice treatment of uUTI. However, three countries (Denmark, Spain, and Scotland) also recommended first-choice antibiotics despite a high prevalence (> 10–20%) of resistant *E. coli*, such as sulphamethizole (resistance rate in Denmark: 37%), trimethoprim (resistance rate in Scotland: 17%, and in Denmark: 10–28%), and fluoroquinolones (resistance rate in Spain: 24%) [14–17,19,21,24].

Use of antibiotics with resistance rates exceeding 10–20% is associated with an increased risk of treatment failure and selection of resistant strains [13,25]. Treatment failure is not common in patients with uUTI, but selection of resistant strains may lead to an increased risk of treatment failures and complications due to resistant bacteria. Despite a sulphamethizole resistance rate of 37% for *E. coli,* this antibiotic is still recommended as first-choice treatment for uUTI in Denmark. Antimicrobial resistance to sulphamethizole has been associated with concurrent resistance to other antibiotics (multidrug resistance), and it should therefore be considered to exclude sulphamethizole in the recommendations for empiric treatment of uUTI [26].

Fluoroquinolones belong to the group of *critically important antibiotics* [6] having an important role in the treatment of more severe infections, such as septicaemia; therefore resistance to fluoroquinolones can have serious clinical consequences. Resistance to fluoroquinolones emerges fast, and it should therefore be used with caution and reserved for severe infections, and preceded by antimicrobial susceptibility testing of the bacteria involved [25].

Susceptibility analyses for *E. coli* have shown resistance rates against amoxicillin/clavulanic acid as high as 29%, when including urine samples from both the primary and secondary healthcare sectors [27]. Furthermore, it has been shown that the increase in resistance to amoxicillin/clavulanic acid is closely related to the resistance to ciprofloxacin, which is as high as 24% in Spain [21,24,28].

Before conclusion, it is important to mention other factors that may influence the increasing resistance to antibiotics. In some countries “over the counter” antibiotics are available at pharmacies. In Spain, for example, Campos et al. found that up to 30% of all antibiotics purchased are “over the counter” and thereby non-prescription [29]. According to a Spanish study carried out in 2008 nearly 80% of the female patients visiting a pharmacy could obtain antibiotics for their UTI without a prescription [30]. Non-prescription antibiotics have been associated with an increase in resistant bacteria in a worldwide study, underlining the need for treatment guidelines, regarding both the diagnosis of infection and the treatment, which is the focus of this article [31].

Another factor affecting resistance is the use of antibiotics in agriculture. In general, resistance to nitrofurantoin, mecillinam, and fosfomycine is low in Europe. This may be due to the fact that these antibiotics are not used in agriculture [32].

## Conclusion

The comparison of different national recommendations for first-choice empiric treatment of uUTI in different European countries showed considerable differences between countries [14–22]. Three countries (Denmark, Scotland, and Spain) recommended empiric treatment with antibiotics despite resistance rates for the most frequent uropathogens exceeding 10–20%. In order to reduce and control the increasing antimicrobial resistance in Europe, it is important to coordinate recommendations for empiric first-choice treatment of uUTI and choose the most appropriate antibiotic with the lowest risk of antibiotic resistance.
